# What is the treatment of tracheal lesions associated with traditional thyroidectomy? Case report and systematic review

**DOI:** 10.1186/s13017-018-0175-4

**Published:** 2018-03-23

**Authors:** Nicola Tartaglia, Roberta Iadarola, Alessandra Di Lascia, Pasquale Cianci, Alberto Fersini, Antonio Ambrosi

**Affiliations:** Department of Surgery, University of Foggia, Luigi Pinto Street, No. 1, 71122 Foggia, Italy

**Keywords:** Total thyroidectomy, Tracheal injury, Tracheal surgery, Tracheal lesion, Tracheal laceration, Tracheostomy

## Abstract

**Aim:**

The aim of this study is to review the literature focusing on various treatments based on time of tracheal injury and on different surgeons’ personal experience.

**Methods:**

We retrospectively reviewed all cases of total thyroidectomy performed at the University Surgical Department of Ospedali Riuniti of Foggia from 2006 to 2017. Only a single case of tracheal lesion due to traditional total thyroidectomy was found. An extensive search of the relevant literature was carried out using MEDLINE (PubMed). We included articles that reported article type, patient number, sex, age, reasons for surgery, time of tracheal perforation intraoperatively or delayed rupture, symptoms, diagnosis, type of surgical procedure, pathological report and follow-up.

**Results:**

A total of 156 published studies were screened from the sources listed. Of these, 15 studies were included in the present study. We introduced our case in the analysis. A total of 16 patients were totally analysed. There were seven males (43.7%) and seven females (43.7%), and for two patients, gender was not available. The mean patient age was 41.6 years.

**Conclusions:**

The literature review showed very few cases treated differently. However, it would be good to standardise treatments. Tracheal perforation, if encountered, needs to be managed appropriately in centres of expertise with a high volume of thyroidectomies.

## Background

Thyroidectomy is one of the most common surgical operations performed in endocrine surgery for benign and malignant thyroid disease. Complication rates from total thyroidectomy are low. At present, mortality for this procedure is approximately 0%, and the overall complication rate is less than 3% [1]. These most commonly include vocal fold paresis or paralysis, hypoparathyroidism, hypocalcaemia, haematoma and wound infection [1, 2]. Tracheal injury associated with thyroidectomy is rare (less than 1%) [3]. The trachea may be perforated or lacerated intraoperatively, often recognised and repaired immediately with little patient morbidity. However, unrecognised injury or delayed rupture secondary to tracheal necrosis can present up to 2 weeks postoperatively [2]. Because this complication occurs in less than one patient for most thyroid surgeons, it is unlikely that any individual will gather sufficient data to report the management in a series [3]. There are no published reports describing the management of inadvertent tracheal perforation during thyroid surgery [3]. Nevertheless, tracheal perforation, if encountered, needs to be managed appropriately. The aim of the present study was to review the literature with a focus on the various treatments based on time of injury and on different surgeons’ personal experience.

## Materials and methods

From 2006 to 2017, approximately 2150 total thyroidectomies were carried out at the University Surgical Department of Ospedali Riuniti of Foggia. We can report only a single case of tracheal injury.

### Literature review

An extensive search for relevant literature was carried out using MEDLINE (PubMed). The keywords used for the search were Tracheal injuries after thyroidectomy, Tracheal injury, Tracheal laceration, Tracheal lesion, used with the Boolean operator ‘AND’ total thyroidectomy. Exclusion criteria were thyroidectomy performed by approaches other than traditional (for example robotic, MIVAT or laser) and other causes of tracheal laceration (for example, perforation due to intubation). Languages were restricted to English and Italian. We included articles that reported article type, patient number, gender, age, reasons for surgery, time of tracheal perforation intraoperatively or delayed rupture, symptoms, diagnosis, type of surgical procedure, pathological report and follow-up.

### Results

A total of 156 published studies were screened from the sources listed. After examination of all titles, 120 papers were excluded as ‘not relevant’. Among the remaining 36 studies, the following were excluded: 4 articles were in other languages, 7 described injury due to thyroidectomy performed by approaches other than the traditional one and 10 analysed other causes of tracheal laceration. Finally, 15 studies were included in the present study (Table 1). We introduced our case in the analysis. A total of 16 patients were analysed. There were seven males (43.7%) and seven females (43.7%), and for two patients, gender was not available. The mean patient age was 41.6 years.

**Table 1:** Literature review and our case

Author	Article type	No. of cases	Reason of TT	Age gender	I setting Y/N	II setting Y/N (day PO)	Size/place trauma	Necrosis/bacterial involvement	Treatment	Posttreatment follow-up
Gosnell [3]	Retrospective 11,917 cases	7/11917 (0.06%)	4 multinodular goitre 1 Hurthle adenoma 1 Grave’s 1 Anaplastic tumour	na	Yes	No	Pl	No	Primarily absorbable sutures with muscle strap in two patients	1 subcutaneous emphysema 1 bilateral pneumothorax
Iacconi [7]	Letter to editor	7	Grave’s disease	na	Yes	No	na	na	na	2 died 1 respiratory distress 2 mediastinitis
Our case	Case report	1	Grave’s disease	44 F	Yes	No	Al	No	Primarily not absorbable sutures with muscle flap	Tracheostomy in VI PO
Damrose [2]	Case report	1	Grave’s disease	20 F	No	Yes (7 days PO)	1–2 mm/A/I ring	No	Primarily absorbable sutures	No
Golger [5]	Case report	1	Grave’s disease	30 F	No	Yes (8 days PO)	A/II-III-IV rings	Yes/*Beta-haemolytic Streptococcus*	Montgomery T-tube with the trap muscles.	T-tube removed 3 months after
Conzo [6]	Case report and review	1	Bilateral adenoma	65 M	No	Yes (15 days PO)	1.5-mm/AL	No	Conservative treatment*	No
Mazeh [8]	Case report and review	1	Grave’s disease	17 F	No	Yes (9 days PO)	2.5-cm/A/II-III-IV rings	No	Conservative treatment*	Primarily suture
Toe Ew [9]	Case report	1	Papillary thyroid carcinoma	62 M	No	Yes (10 days PO)	A	No	na	na
Jacqmin [10]	Case report	1	Grave’s disease	53 F	No	Yes (8 days PO)	A/III ring	Yes/*Streptococcus mitis*	Circumferential tracheal excision with anastomosis	No
Chauhan [11]	Case report	1	Medullary carcinoma thyroid	65 M	No	Yes (7 days PO)	5 mm/A II ring	Yes	Debridement tracheostoma drain	Chauhan [11]
Benjamin [12]	Case report	1	Papillary thyroid carcinoma	55 M	No	Yes (4 days PO)	na	na	Primarily absorbable sutures	No
Bertolaccini [13]	Case report	1	Papillary thyroid carcinoma	45 M	No	Yes (4 days PO)	AL/IV ring	*Staphylococcus aureus*	Primarily absorbable sutures with muscle flap	No
Sanna [4]	Case report and review	1	Papillary thyroid carcinoma	17 F	No	Yes (7 days PO)	3 cm/AL/II–V rings	No	A muscular flap, rounded on the tracheal defect	No
Rosato L [14]	Case report	1	na	na	No	Yes (10 days PO)	na	na	Primarily sutures with patch of fibrinogen-thrombin collagen	No
Escott [15]	Case report	1	na	29 M	No	Yes (14 days PO)	2.5-mm PL	na	Conservative treatment*	After 48 h with a myovascular transposition flap with Tisseel tissue-bonding agent.
Xinwei Han [16]	Case report	1	Nodule	39 F	no	Yes (1 PO)	na	na	Covered tracheal stent	Abscess removed with drainage stent was removed 55 days after

*F* female, *M* male, *TT* total thyroidectomy, *L* lymphadenectomy, *PR* primarily repair, *MF* muscolar flap, *D* drains, *PO* postoperative, *I setting* trachea may be perforated or lacerated intraoperatively, *II setting* unrecognised injury, or delayed rupture secondary to tracheal necrosis, can present up to 2 weeks postoperatively, *PL* posterolateral surface of trachea, *AL* anterolateral, *A* anterior, *na* not applicable

*Conservative treatment (bed rest, antibiotic and cough suppressants)

## Case report

**Fig. 1:**
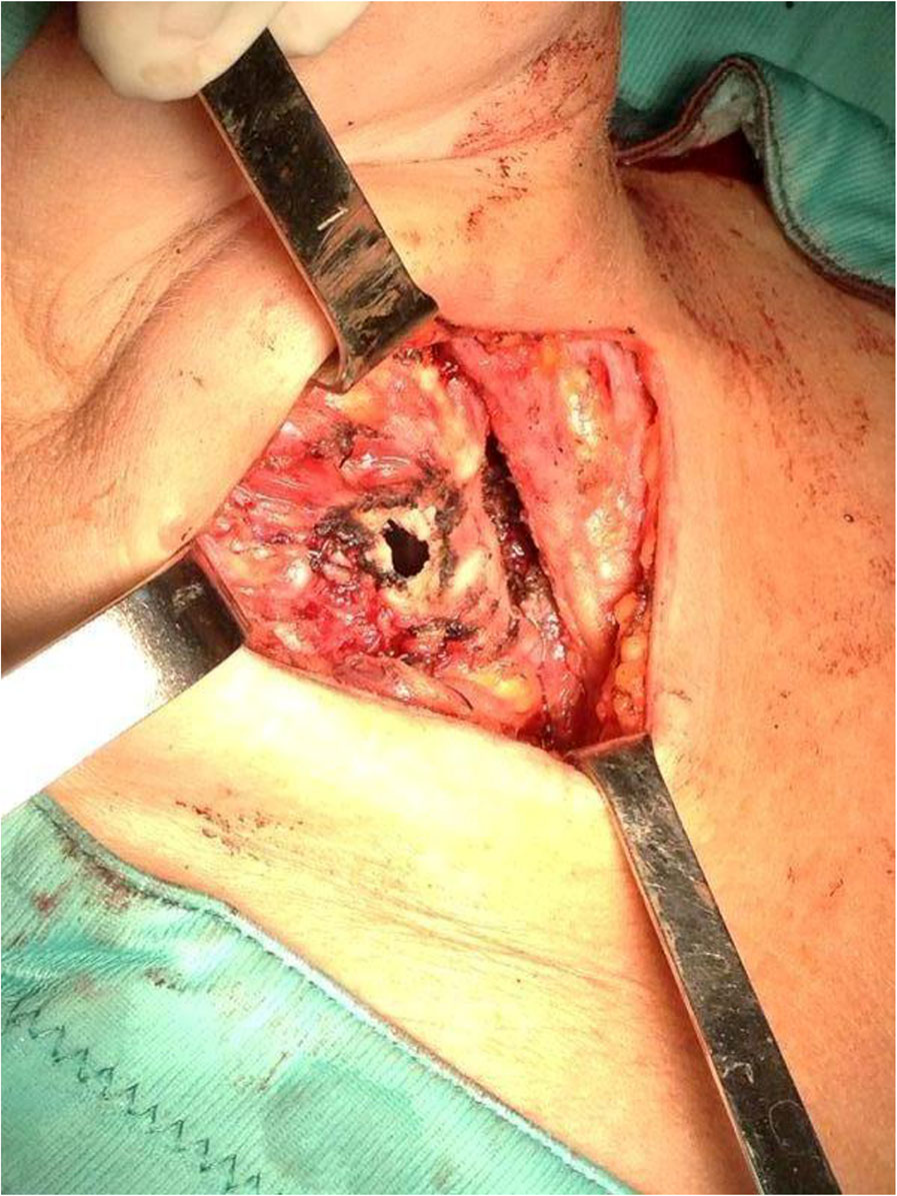
Trachea injury identified during the surgery

A 44-year-old Romanian woman underwent elective total thyroidectomy in our department for Grave’s disease. Euthyroidism was preoperatively achieved with methimazole and Lugol’s solution. At the time of the surgical procedure, due to Grave’s disease, a fibrotic and vascular thyroid gland was found. Total thyroidectomy was performed with the use of a combination of electrocautery and shears. During mobilisation of the thyroid gland, all four parathyroid glands and both recurrent laryngeal nerves were identified and carefully preserved. Because of the fibrotic nature of the gland, the use of a bipolar device (Enseal) was necessary for management of the right Gruber ligament. Immediately after, thermal damage to the anterior-lateral surface of the trachea (I/II tracheal ring) was found (1.5 cm, Fig. 1). This was repaired primarily using non-absorbable sutures (Prolene 3/0). A muscle flap was used for reinforcement, and a suction drain was left at the surgical site. The patient was subsequently transferred to intensive care for delayed extubation that occurred on postoperative (PO) day 5. On the same day, because of her respiratory condition, the patient underwent bronchoscopy and subsequent tracheostomy because the wound remained open. On PO day 7, she was readmitted to our department with tracheostomy, spontaneously breathing and with good cardio-circulatory parameters. The ear, nose and throat consultant diagnosed adduction vocal cord paralysis for which she started phonation and swallowing training. The patient was discharged on PO day 26, euthyroid on substitution treatment. At follow-up, 6 months later, she had no vocal cord paralysis. However, she still had the tracheostomy. Subsequently, the patient returned to her country for personal reasons and was lost to follow-up.

## Discussion

Complication rates from total thyroidectomy are low. At present, mortality for this procedure is approximately 0%, and the overall complication rate is less than 3% [1, 2, 4]. Major complications include wound infection (0.02–0.5%), haematoma (0.3–4.3%), transient recurrent laryngeal nerve palsy (1–2%), permanent recurrent laryngeal nerve palsy (< 1%), transient hypoparathyroidism (1.6–50%) and permanent hypoparathyroidism (0–13%) [1]. During the period between 1997 and 2016, we performed 2150 thyroidectomies with the following percentage of complications: wound infection (0.002%), haematoma (1.3%), transient recurrent laryngeal nerve palsy (1.5%), permanent recurrent laryngeal nerve palsy (0.2%), transient hypoparathyroidism (5.9%) and permanent hypoparathyroidism (0.3%).

### Risk factors

It is possible to identify several preoperative, intraoperative and postoperative risk factors. Female gender and thyrotoxic goitre are commonly considered risk factors [2]. Golger et al. [5] and Conzo et al. [6] suggest that long-term tracheal compression by a large goitre may cause local tracheal wall weakening and subsequent tracheomalacia. Intraoperative risk factors include prolonged intubation and elevated cuff pressure because of reduction of blood supply to the trachea with subsequent damage [2]. Gosnell et al. [3] report frequent exceeding difficulty to define the plane of dissection in patients with multi-nodular goitre that is characterised by repeated cycles of hyperplasia, degeneration and fibrosis. These often contain dense fibrotic thyroid tissue that is contiguous with surrounding fibrous tissues, including the trachea. This condition is associated, as in our case, with considerable use of a device. The use of a device for diathermy can be dangerous with respect to tracheal lesions. When dissecting around the trachea, the lateral pedicles should be carefully preserved to maintain the blood supply to the upper segments, and thyroid branches should be ligated close to the capsule. Furthermore, extended operating time always increases the risk of complications [5]. In addition, Lugol’s solution may reduce the amount of intraoperative cautery required for control of haemorrhage. In the postoperative period, persistent uncontrolled cough can be another risk factor.

A review of the literature identified two distinct settings for tracheal injury associated with thyroid surgery (Table 1). In the first setting, the trachea may be perforated or lacerated intraoperatively. In the second, unrecognised injury or delayed rupture secondary to tracheal necrosis can present up to 2 weeks postoperatively.

#### First scenario: trachea injury identified during the surgery

To our knowledge, we described the third case reported in the literature [3, 7]. In this setting, a full-thickness thermal injury to the trachea can occur and is typically identified when the endotracheal tube is observed through the tracheal defect. This type of tracheal injury is usually associated with either excision of large and bulky tumours or technical errors and is almost always repaired primarily, with no further consequences [8]. Assessment of the actual rate of such tracheal injuries is difficult because they are underreported. In one study, Gosnell et al. [3] reviewed 11,917 thyroidectomies performed during a 45-year period and identified tracheal perforations in 0.06%. In that report, all perforations were located at the posterolateral surface of the trachea, either following attempted suture ligation of vessels in the region of the ligament of Berry or with the use of diathermy adjacent to the trachea (Table 1). The same author reported that most perforations were recognised as they occurred, but smaller inadvertent injuries were identified at the conclusion of the procedure. Because visual inspection of the trachea may miss small perforations when an injury is suspected during the surgical procedure, saline surrounding the trachea may be used to test for an air leak. This is more sensitive and is recommended routinely for all thyroid surgeries [2, 3]. Once identified, the defect must be repaired by single absorbable suture [3] or non-absorbable. Subsequently, a muscle flap can be used for reinforcement. Placement of drains and the use of postoperative antibiotics are dictated by surgeon preference.

#### Second scenario: trachea injury identified after the surgery

The second setting, which includes delayed tracheal rupture after a thyroid surgical procedure, seems to be less rare, but it too has only been described in case reports [2, 4–6, 8–16]. As reported by Mazeh et al. [8], in this setting, the thermal injury during the initial procedure is not recognised by the surgeon, and over the course of several days, tracheal necrosis surrounding the thermal injury results in an air leak to the surrounding tissues [8]. These can lead to extensive subcutaneous and mediastinal emphysema and severe dyspnoea. It can also adversely affect venous return to the heart. In addition, tracheal rupture may cause neck infections, which may lead to mediastinitis, mediastinal abscess and death [16, 17]. In addition to the above mentioned generic risk factors, the damage to the tracheal wall and possible development of a haematoma may create an environment in which bacteria can seed, resulting in infection and necrosis [5]. Careful elicitation of history may reveal strenuous coughing or sneezing as a precipitating factor [8]. Typically, these patients present on varying postoperative days with either subcutaneous emphysema or wound infection. Other symptoms may include neck or facial swelling, haemoptysis, retrosternal pain or hoarseness [2, 5]. Respiratory compromise may follow. The diagnosis is clinical and should be confirmed with a CT scan that rules out other causes of subcutaneous emphysema (such as spontaneous pneumothorax). CT may demonstrate the tracheal defect and provide localization. Of importance, a normal tracheal outline does not exclude the diagnosis [8]. Flexible or rigid bronchoscopy may also locate the tracheal rupture. Nevertheless, some injuries may not be identified [2, 10]. Airway control should be a priority. Equipment should be on hand for performing an emergent tracheostomy if necessary. Rapid sequence induction of anaesthesia and orotracheal intubation with placement of the cuff distal to the site of injury is usually sufficient to control the airway [13]. The management of delayed tracheal rupture after thyroidectomy should not differ from that of any other delayed tracheal injury. Despite past assumptions that surgical treatment is indicated in all cases, recent studies have identified a subset of patients who could and should be managed conservatively [18, 19]. If necessary, surgery must not be delayed [13]. Potential patient morbidity can include neck abscess, mediastinitis, pneumothorax, cardiac tamponade and tension pneumomediastinum. Mortality has been reported [2]. By review of literature data in this setting, we found 15 case reports [2, 4–6, 8–16] (Table 1). Five case reports reported patients affected by Grave’s disease. The other five patients were affected by malignant disease of the thyroid gland and only one by bilateral adenoma. The diagnosis of tracheal injury occurred between the 1st and 15th postoperative day [6, 16]. In most of these patients, necrosis was found, and culture revealed bacterial infection in 3/15 patients by *Beta-haemolytic Streptococcus* [5], *Streptococcus mitis* [10] and *Staphylococcus aureus* [13]. The management depends on the authors and was very different among them. In all cases where necrosis was present, debridement of the devitalized trachea was necessary. Primary conservative treatment (bed rest, antibiotic and cough suppressants) was attempted by Conzo et al. [6] with success and by Mazeh et al [8] with failure. The first [6] was a very small laceration (1.5 mm). Primary closure of the wound with absorbable suture, with or without drains, was attempted first by different authors [2, 12] and Mazeh et al. [8] after conservative treatment failure. Bertolaccini et al [13] and Sanna et al [4] opted for a muscle flap stitched with an absorbable suture on the defect. There was no necrosis or infection in these patients, and this procedure was successful. Other authors reported good results with primary sutures with a patch of fibrinogen-thrombin-coated collagen [14] or a myovascular transposition flap in conjunction with a Tisseel tissue-bonding agent after conservative treatment failure [15]. In contrast, large extension of a tracheal tear and a wide necrosis or infection contraindicated a primary repair. Golger et al [5], after debridement, chose to partially close the trachea around a no. 11 Montgomery T-tube with adjacent strap muscles that were removed after 3 months. Jacqmin et al [10] preferred circumferential tracheal excision with anastomosis. Chauhan et al [11], after debridement, chose tracheostomy (removed 14 days postoperative) and a drain. Finally, Han et al. [16] proposed a tracheal stent that was implanted to cover the entire lesion complicated by abscess. Use of a covered tracheal stent for treatment of tracheal rupture represents a minimally invasive technique that can be performed under local anaesthesia by interventional radiologists. The procedure is likely to be especially suited to patients with poor general condition. The implantation can be performed quickly (usually 3–5 min) and results in effective and instant coverage of the site of tracheal rupture.

Some authors try to identify a precise treatment based on the size of the lesion and the clinical condition of the patient. As reported by Mazeh et al [8], patients with no respiratory distress who have stable or improving symptoms and no evidence of infection may be observed as in-patients while receiving intravenous antibiotic coverage. An aggressive surgical approach is warranted in all other patients. Other authors proposed different criteria. Kaloud et al. [20], for example, reported that surgical repair is indicated for transmural tears with a length exceeding 1 cm. In contrast, Kuhne et al. [21] reported that conservative treatment may be indicated for tracheal disruptions less than 2 cm. Large extension of the tracheal tear and wide necrosis or infection contraindicated primary repair. In several case reports, the authors used cultures obtained from the neck wound intraoperatively to guide future antibiotic coverage. When possible, primary repair should be attempted over viable tracheal edges. Repairs may be reinforced with muscle flaps, and a suction drain may be left at the surgical site for possible future ongoing leaks. The rationale for use of a muscle flap is to reinforce the repair with viable muscle tissue, which may help to prevent postoperative leaks. This tissue plastically adheres to the airway lesion and permits to maintain its rigidity, obtaining a good result. This potentially prevents further complications because of its vascular feeding [8, 13]. No published studies have assessed the utility of such a flap. Similarly, the use of suction drains is not supported by any high-quality studies [22].

## Conclusion

Tracheal injury associated with thyroidectomy is very rare, but when it occurs, it can be dangerous. Tracheal perforation is generally not considered a complication as such but rather a technical occurrence during surgery that requires expeditious attention. A literature review revealed very few cases treated in different ways. Nevertheless, it would be good to standardise the treatments. Tracheal perforation, if encountered, needs to be managed appropriately in centres of expertise with a high volume of thyroidectomies.
